# African Swine Fever Virus Ubiquitin-Conjugating Enzyme Interacts With Host Translation Machinery to Regulate the Host Protein Synthesis

**DOI:** 10.3389/fmicb.2020.622907

**Published:** 2020-12-15

**Authors:** Lucía Barrado-Gil, Ana Del Puerto, Raquel Muñoz-Moreno, Inmaculada Galindo, Miguel Ángel Cuesta-Geijo, Jesús Urquiza, Estanislao Nistal-Villán, Carlos Maluquer de Motes, Covadonga Alonso

**Affiliations:** ^1^Department of Biotechnology, Instituto Nacional de Investigación y Tecnología Agraria y Alimentaria (INIA), Madrid, Spain; ^2^Microbiology Section, Departamento Ciencias Farmacéuticas y de la Salud, Facultad de Farmacia, Instituto de Medicina Molecular Aplicada (IMMA), Madrid, Spain; ^3^Department of Microbial Sciences, School of Biosciences and Medicine, University of Surrey, Guildford, United Kingdom

**Keywords:** ubiquitin-conjugating enzyme, viral E2, ribosomal protein 23, translation initiation factor, eIF4E, African swine fever virus, ASFV, Cullin 4B Cul4B

## Abstract

African Swine Fever virus (ASFV) causes one of the most relevant emerging diseases affecting swine, now extended through three continents. The virus has a large coding capacity to deploy an arsenal of molecules antagonizing the host functions. In the present work, we have studied the only known E2 viral-conjugating enzyme, UBCv1 that is encoded by the *I215L* gene of ASFV. UBCv1 was expressed as an early expression protein that accumulates throughout the course of infection. This versatile protein, bound several types of polyubiquitin chains and its catalytic domain was required for enzymatic activity. High throughput mass spectrometry analysis in combination with a screening of an alveolar macrophage library was used to identify and characterize novel UBCv1-host interactors. The analysis revealed interaction with the 40S ribosomal protein RPS23, the cap-dependent translation machinery initiation factor eIF4E, and the E3 ubiquitin ligase Cullin 4B. Our data show that during ASFV infection, UBCv1 was able to bind to eIF4E, independent from the cap-dependent complex. Our results provide novel insights into the function of the viral UBCv1 in hijacking cellular components that impact the mTORC signaling pathway, the regulation of the host translation machinery, and the cellular protein expression during the ASFV lifecycle.

## Introduction

African swine fever virus (ASFV) is a large, enveloped virus from the *Asfarviridae* family, with icosahedral morphology and an average diameter of 200 nm. The viral genome consists of a single molecule of linear, double-stranded DNA with covalently closed ends and different genome sizes ranging from 170 to 190 Kbp depending on the viral isolate. ASFV is the causative agent of African swine fever (ASF), one of the most relevant diseases of swine that is associated with an important socioeconomic burden and it is currently spreading widely throughout Asia, Europe, and Africa (Zhou et al., 2018; Garigliany et al., 2019). It has been reported for the first time in dozens of countries and very recently in Germany (Friedrich-Loeffler-Institut, 2020). Currently, there is not any commercial vaccine available and the only control measure is the culling of infected animals.

Ubiquitin (Ub) is a small and highly conserved protein present in all eukaryotic cells. The covalent attach of these few amino acids to lysine residues of the target protein is called ubiquitylation (Thrower et al., 2000). Ubiquitylation of proteins is relevant for a wide variety of cellular processes (Mosesson et al., 2003). The conjugation of ubiquitin to its substrates involves three sequential steps. First, the ubiquitin-activating enzyme (E1) forms a thiol ester bond with the C-terminal Gly of ubiquitin. Activated ubiquitin is then transferred to an ubiquitin-conjugating enzyme (E2) by transesterification. E2 are responsible of initiation and elongation, regulate the formation and establish the topology of the assembled Ub chains. In turn, ubiquitin will be attached to the substrate protein through an ubiquitin ligase (E3). This last step is critical for the specificity and the efficiency of the reaction (Schulman and Harper, 2009).

The chain length (poly- vs. monoubiquitylation) as well as the lysine residue used for chain elongation are critical factors to determine the fate of an ubiquitylated protein. Ub chains formed through Lys-48 (K48) or Lys-63 (K63) are typically involved in proteasomal degradation and signal transduction, respectively. However, Ub can be conjugated through other Lys residues such as K6, K11, K27, K29, and K33, providing Ub chains of different lengths, shapes, and roles, of mostly unexplored functions (Komander and Rape, 2012).

ASFV is the only virus that is known to encode for an ubiquitin-conjugating enzyme (from now on referred to as UBCv1) or E2, which is the product of ASFV gene *I215L* (Hingamp et al., 1992; Rodriguez et al., 1992). UBCv1 is an early viral protein (Yanez et al., 1995) with nuclear and cytoplasmic distribution that can be found in the viral factories (VFs) from 8 hpi (Freitas et al., 2018). Also, UBCv1 transient knockdown using siRNA impairs viral infection (Freitas et al., 2018). ASFV UBCv1 shares a 30–48% amino acid identity to cellular E2 enzymes. The C-terminal extensions of cellular E2s are variable in length but similar to ASFV UBCv1 in the high acidic residues content (Hingamp et al., 1992). Indeed, mutagenesis studies have shown that UBCv1 C-terminal acidic extension is required for nuclear accumulation (Bulimo et al., 2000). This viral protein is polyubiquitylated and its catalytic site Cys85 has an important functional role (Freitas et al., 2018). Ubiquitylation of some viral proteins such as the product of the ASFV gene *PIG1* is UBCv1-dependent and does not require E3 activity (Hingamp et al., 1995). To date, the only described *in vitro* binding partner of UBCv1 is the protein SMCp, similar to the ARID (A/T rich interaction domain) family, of unknown significance (Bulimo et al., 2000).

Ubiquitylation plays an important role in host translation by regulating the ribosome-associated quality control (Juszkiewicz and Hegde, 2017). It is well known that viruses have developed mechanisms to control the cellular translation activity to favor the synthesis of their proteins. Most of these strategies consist on switching on/off the activity of key eukaryotic translation initiation factors (eIFs) essential for the host protein synthesis (Schneider and Mohr, 2003). Viruses have developed multiple strategies to hijack eIFs promoting viral over cellular translation. A key factor is eukaryotic translation initiation factor 4F complex (eIF4F), which is composed of three proteins: the eukaryotic translation initiation factor 4A (eIF4A), 4E (eIF4E), and 4G (eIF4G) (Prevot et al., 2003). eIF4E binds to the m7GpppG 5′ terminal “cap” structure of mRNA and eIF4G is a scaffold protein between the mRNA and the small ribosomal subunit 40S. eIF4E is also involved in the recruitment of the ribosome to the mRNA cap structure, a critical function in the regulation of translation initiation (Uttam et al., 2018) that is tightly regulated by post-translational modifications. The eIF4E-binding proteins (4E-BPs) are well-known translational repressors of the cap-dependent translation (Sonenberg and Hinnebusch, 2009). In their hypo-phosphorylated state, 4E-BPs can bind and hijack the eIF4E factor, preventing their binding to eIF4G, which alters the assembly of the eIF4F complex. 4E-BPs can be inactivated by mTOR-mediated hyperphosphorylation, thus preventing eIF4E binding and ultimately allowing cap-dependent translation (Bhandari et al., 2001; Richter and Sonenberg, 2005). Interestingly, and similar to Vaccinia virus (VACV) and other DNA viruses, it has been described that ASFV infection promotes 4E-BP1 phosphorylation at early but not late times post-infection (Buchkovich et al., 2008; Castello et al., 2009).

In this work, we have studied UBCv1, the unique viral E2 ubiquitin-conjugating enzyme and its cellular binding proteins. Proteomic analysis in combination with an alveolar macrophage library screening allowed us to identify previously uncharacterized interactions of UBCv1 with the 40S ribosomal protein RPS23, traslation initiation factor eIF4, and the cellular ligase Culin 4B. Our findings revealed that the UBCv1 protein can impact the mTORC signaling pathway.

## Materials and Methods

### Cell Culture

Vero (ATCC CCL-81; renal fibroblasts), Cos-7 (ATCC CRL-1651, Richmond, VA, United States), and HEK293T (ATCC CRL-11268) cell lines were maintained in Dulbecco’s modified Eagle medium (DMEM) containing 100 U/ml penicillin, 100 g/ml streptomycin, 2 mM GlutaMAX and supplemented with 5 or 10% of heat-inactivated fetal bovine serum (FBS). FBS was reduced to 2% in the inoculum at the time of viral adsorption and throughout the infection process. All these mammalian cells were grown at 37°C and 5% CO_2_ conditions. Sf21 (PLB-SF21-AE) cells were cultured at 27°C in TNMFH medium with 10% FBS and gentamicin (50 μg/ml).

### Viruses and Infection

We used the cell culture-adapted and non-pathogenic ASFV isolate Ba71V (Enjuanes et al., 1976), the recombinant Ba71V-30GFP (BPP30GFP) (Barrado-Gil et al., 2017) and Ba71V-Bp54GFP (B54GFP-2) (Hernaez et al., 2006) isolates. ASFV viral stocks were propagated and titrated by plaque assay in Vero cells, as previously described (Enjuanes et al., 1976). When using the recombinant viruses BPP30GFP or B54GFP-2, green fluorescent plaques were observed 4 days after infection under the fluorescence microscope. ASFV stocks were partially purified using a sucrose cushion (40%) in PBS at 68,000 × *g* for 50 min at 4°C and were further used at a multiplicity of infection (moi) of 1 unless otherwise indicated.

### Antibodies

The following rabbit antibodies were used: Flag (Sigma-Aldrich, St Louis, MO, United States), c-Myc-HRP (Miltenyi Biotec, North Rhine-Westphalia, Germany); RPL11 (D1P5N), Phospho-4EBP1 (Thr70), 4EBP1, S6 ribosomal protein (5G10), Phospho-S6 ribosomal protein (Ser235/236) and TSC2 (Cell Signaling, Danvers, MA, United States) and HA-HRP (Thermo Fisher Scientific, Waltham, Massachusetts, United States). Mouse monoclonal antibodies used were: HA, c-Myc, RPS23 (1E3) and Tubulin (B512) (Sigma-Aldrich, St Louis, MO, United States); eIF4E (BD Biosciences, Franklin Lakes, New Jersey, United States); p72 (1BC11), p72 (18BG3), and p150 (17AH2) (Ingenasa, Madrid, Spain); Cullin4B and RPS23 (Abcam, Cambridge, United Kingdom) and p30 (a gift from J.M. Escribano, Algenex, Madrid, Spain). Horseradish peroxidase-conjugated anti-rabbit and anti-mouse secondary antibodies were from Sigma-Aldrich (St Louis, MO, United States), and Alexa-Fluor−488, −594, and −647 conjugated anti-rabbit and anti-mouse antibodies were from Thermo Fisher Scientific (Waltham, Massachusetts, United States).

### Plasmids and Transfections

Wild type UBCv1 (UBCv1) and a cysteine 85 mutant (UBCv1C85A) were cloned into a pcDNA4/TO plasmid fused to an N-terminal 3XFlag (kindly provided by C. Maluquer de Motes). Primers used to generate these constructs are described in Table 1. For the ubiquitylation assay, we used several pcDNA3.1 hemagglutinin-(HA)-tagged ubiquitin (Ub) plasmids (Gack et al., 2007).

**TABLE 1:** Primers used in cloning techniques.

Name	Sequence (5′—3′)
I215L/Flag_Fw	GCGCGCGGCCGCAGTTTCCAGGTTTTTAATAGC
I215L/Flag_Rev	GCGCTCTAGATTACTCATCATCCATCTCTTCATC
I215L-mut_Fw	CCCTGATGGAAGACTAGCAATCTCTATCTTACACG GAGACAATGC
I215L-mut_Rev	GCATTGTCTCCGTGTAAGATAGAGATTGCTAGTC TTCCATCAGGG
I215L/pFB_Fwd	GCGCGGATCCATGGTTTCCAGGTTTTTAATAG
I215L/pFB_Rev	GCGCTCTAGACTCATCATCCATCTCTTCATCC

Cell transfections were carried out with Lipofectamine 2000 (LF2000, Invitrogen) following the manufacturer’s instruction. Then, 16–24 h after transfection, cells were harvested and analyzed through different methodologies.

### Generation of UBCv1 Polyclonal Antibody

The 648-bp sequence encoding the viral UBCv1 protein was PCR-amplified using Ba71V purified DNA as a template (Table 1). Then, it was subcloned into a modified pFastBac1 (pFB) vector previously generated in our lab (Okamoto et al., 1999; Gomez-Sebastian et al., 2014). The recombinant baculoviruses (rBacs) were obtained by generating the bacmids using the pFB-UBCv1-His-KDEL vector for use with the Bac-To-Bac baculovirus expression system (Invitrogen, Life Technologies). Bacmids were transfected into Sf21 cells using CellfectinHII Reagent (Invitrogen, Life Technologies) and following the manufacturer’s instructions. After 72 h at 28°C, rBacs expression was confirmed by western blot (WB). Then, we scaled up the production of rBacs and the recombinant protein was purified in a resin column using α-His-HRP antibody and finally eluted with imidazole. A collaboration with the biotech company Algenex was established to scale up the production of purified protein by infecting Lepidoptera Trichoplusia ni (T. ni) pupae. These pupae were infected with our rBacs and incubated for 96 h at 28°C and subsequently frozen and lysed. The expression of UBCv1 in these pupae was tested before protein purification. The total amount of purified UBCv1 (1 mg/ml) was injected in rabbits for the generation of a polyclonal serum against the viral protein UBCv1 (Protein Alternatives SL, PROALT). The antiserum was validated by WB and IF.

### Chemical Reagents

Proteasome inhibitors MG132 (Calbiochem), bortezomib (Bort) (Santa Cruz Biotechnology), and lactacystin (Lact) (Santa Cruz Biotechnology) were dissolved in dimethyl sulfoxide (DMSO) and used at a concentration of 1, 20, and 0.5 μM, respectively. Endosomal acidification inhibitor bafilomycin (Baf) (Sigma Aldrich), dynamin-inhibitor dynasore (Dyn), and inhibitor of Na + H + exchanger EIPA were dissolved in DMSO and used at 0,2, 80, and 50 μM, respectively. PI3K inhibitors wortmanin (Wort) and LY294002 (LY294) and E1 inhibitor Pyr-41 were dissolved in DMSO and used at 10 μM. DNA synthesis inhibitor AraC and protein synthesis inhibitor cycloheximide (CHX), were dissolved in double-distilled H_2_O (ddH_2_O) and used at 100 μg/ml. Working solutions at the indicated concentrations were freshly prepared in DMEM supplemented with 2% FBS. Before infection, cells were pretreated with these drugs for 1 h except for Pyr-41 where cells were pre-treated for 8 h. Drugs were present throughout the course of the experiment unless otherwise indicated. To determine the aforementioned working concentrations, we analyzed cell viability and cytotoxicity tests for each drug treatment with CellTiter 96 Non-radioactive Cell Proliferation Assay (Promega) and following the manufacturer’s instructions. Based on these results, we selected the optimal non-toxic concentrations for setting up all the experiments.

### Protein Extraction and Electrophoresis Assay

Protein extracts from uninfected or infected cells we collected using Laemmli 2x Buffer (BioRad). For standard transfection assays, cells extracts were harvested in RIPA buffer (50 mM TrisHCl pH 7.4, 1 mM EDTA, 1 mM EGTA, 100 mM NaCl, 1% Triton X100, 0.2% sodium deoxycholate, and 0.1% SDS) supplemented with 1X protease inhibitors (complete Mini, EDTA free protease inhibitor cocktail tablets, Roche) and 1X phosphatase inhibitors (PhosSTOP EASY pack, Roche). For mass spectrometry samples, 0.5% NP-40 buffer was used: 0.5% of Non-idet P40 substitute (Sigma Aldrich) in PBS supplemented with 1X protease and phosphatase inhibitors as above.

Protein lysates were separated based on electrophoretic mobility in sodium dodecyl sulfate polyacrylamide gels (SDS-PAGE) under reducing conditions and transferred to nitrocellulose membranes (Amersham). Membranes were blocked with 5% of non-fat milk in PBS−0.05% Tween-20 (Sigma Aldrich) and further incubated with both primary and horseradish peroxidase-conjugated secondary antibodies. Protein expression was analyzed using the molecular imager Chemidoc XRSplus Imaging System. Bands were quantified by densitometry and normalized using the Image Lab software (BioRad).

### Immunofluorescence

Vero cells were seeded at a variable density onto 12 mm glass coverslips in 24 well plates before infection, transfection, or drug treatment. Then, cells were washed with PBS and fixed with 4% paraformaldehyde (PFA) for 15 min. After washing with PBS, cells were permeabilized with 0, 1% Triton X-100 in PBS for 10 min. Then, coverslips were washed with PBS and incubated in 2% bovine serum albumin (BSA, Sigma) diluted in PBS for 1 h. Slides were then incubated at room temperature for 1 h in primary antibody diluted in PBS-BSA 1%. Appropriate secondary antibodies conjugated to either Alexa Fluor-488, -594, or -647 (Thermo Fisher Scientific) were used and cell nuclei were detected with TOPRO3 (Thermo Fisher Scientific). Coverslips were mounted on glass slides using ProLong Gold (Thermo Fisher Scientific). Cells were visualized using a TCS SPE confocal microscope (Leica) and data were analyzed using Leica Confocal Software and ImageJ.

### Ubiquitylation Assay

To examine ubiquitylation, 60 mm plate HEK 293T cells were transfected with 3XFlag-UBCv1, UBCv1mut, or HA-tagged Ub plasmids and further lysed in TNE buffer 24 h later as previously described (Yamazaki et al., 2009). Samples were centrifuged (15,000 × *g* for 30 min) and supernatants were mixed with 1 volume of 2% SDS TNE. Samples were boiled at 90°C for 10 min to eliminate non-covalent interactions and lysates were 10-fold diluted in TNE buffer and FLAG-immunoprecipitated using a FLAG M2 resin (Sigma-Aldrich). Samples were then washed three times in TNE buffer and analyzed by WB.

### Yeast-Two Hybrid

pGBT9-UBCv1 was used to screen a pACT2 cDNA library from swine alveolar macrophages in *Saccharomyces cerevisia*e reporter strain Y190 (Miskin et al., 1998). The alveolar macrophage library used for screening λ-ACT2 Library (3.6 × 10^6^) clones was a kind gift from Dr. Dixon (The Pirbright Institute, United Kingdom). The two types of hybrid plasmids were sequentially co-transformed into Y190 reporter yeast host strain with the lithium acetate (LiAc) procedure (Gietz et al., 1992). Clones encoding interacting proteins were selected on medium lacking histidine and by expression of β-galactosidase (β-gal) (Chien et al., 1991). All positives transformants were then tested and segregated three times to eliminate false positives. Finally, the coding sequences contained in the two clones that were considered as positives were amplified with pACT2 specific primers, sequenced, and matched in the NCBI database.

### Ribosome Fractionation

Monolayers of Vero cells mock-infected or ASFV-infected were treated with 100 μg/ml of cycloheximide (CHX) for 10 min at 37°C. At 48 hpi, cells were harvested, centrifuged at 680 × *g* for 5 min, washed twice with PBS supplemented with 100 μg/ml of CHX, and resuspended in 1X polysome buffer (PB) (200 mM Tris pH 7.4–7.5, 100 mM NaCl, 30 mM MgCl_2_, 100 μg/ml CHX). Samples were kept on ice for a few minutes and the lysates were further homogenized in a detergent-containing buffer (1.2% Triton-X100, 200 mM sucrose, 100 μg/ml CHX, dissolved in 1X PB) and using a Dounce homogenizer. Samples were centrifuged at 12,500 x *g* for 10 min and supernatants were mixed with Heparin solution (10 mg/ml Heparin and 1.5 M NaCl in 1X PB). To isolate the polysome fraction, 15–50% sucrose gradients were created by mixing 15 and 50% sucrose solutions (sucrose dissolved in 1X PB). Samples were loaded on top of the sucrose gradients and ultracentrifuged for polysome separation at 200,000 × *g* for 2 h at 4°C in a SW40 Ti rotor (Beckman Coultier). Finally, fractions of 1 ml were collected and precipitated using the methanol/chloroform protein precipitation method. Four volumes of ethanol, one volume of chloroform and three volumes of ddH_2_O per volume of sample were added.

### Immunoprecipitation (IP)

HEK 293T cells were transfected with pcDNA4/TO, pcDNA4/TO-UBCv1, or pcDNA4/TO-UBCv1C85A using polyethylenimine (PEI) or Lipofectamine 2000 (LF2000) transfection reagent and following manufacturer’s instructions. After 24 h, cells were washed once with ice-cold PBS and lysed with IP buffer [10% glycerol, 10 mM CaCl2, 150 mM NaCl, 20 mM Tris-HCl (pH 7.4), 0.1% Triton-X100, and proteases/phosphatases inhibitors (Roche)]. After centrifugation (15,000 × *g* for 20 min), supernatants were incubated with Flag M2 resin (Sigma Aldrich) at 4°C for 16 h. After three washes with ice-cold IP buffer, beads were boiled and analyzed by WB.

### SILAC Quantitative Proteomics

SILAC quantitative proteomics was performed as previously described (Odon et al., 2018) with minor differences. HEK293T cells were cultured for at least 5 times in Arg/Lys-free MEM supplemented with Pen/Strep, dialyzed FCS, and either unlabeled or stable isotope-labeled forms of Arg and Lys (DC Biosciences). Cells were transfected with 10 μg of pcDNA/TO-3XFlag-UBCv1 using PEI and harvested 24 h later in PBS supplemented with 0.5% NP-40 (Sigma) and protease and phosphatase inhibitors (Roche). The lysates were incubated 20 min in ice and centrifuged at 15,000 × *g* for 20 min at 4°C. Cleared lysates were normalized for total amount protein using bicinchoninic acid (BCA) protein assay (Pierce) and subjected to Flag immunoprecipitation as described above. Denatured eluates were combined on a 1:1 ratio and subjected to in-gel tryptic digestion using a ProGest automated digestion unit (Digilab United Kingdom). The resulting peptides were fractionated using an Ultimate 3000 nanoHPLC system in line with an Orbitrap Fusion Tribrid mass spectrometer (Thermo Fisher Scientific). All spectra were acquired using Xcalibur 2.1 software (Thermo Fisher Scientific) and operated in data-dependent acquisition mode. FTMS1 spectra were collected at a resolution of 120 000 over a scan range (m/z) of 350–1,550, with automatic gain control (AGC) target of 300 000 and a max injection time of 100 ms. Precursors were filtered using an Intensity Range of 1E4–1E20 and according to charge state (to include charge states 2–6) and with monoisotopic precursor selection. Previously interrogated precursors were excluded using a dynamic window (40 s ± 10 ppm). The MS2 precursors were isolated with a quadrupole mass filter set to a width of 1.4 m/z. ITMS2 spectra were collected with an AGC target of 20 000, max injection time of 40 ms, and CID collision energy of 35%.

### Mass Spectrometry Data Analysis

The raw data files were processed and quantified using Proteome Discoverer software v1.4 (Thermo Fisher Scientific) and searched against the UniProt Human database (downloaded 29/06/17; 140,000 entries) plus the ASFV I215 protein sequence using the SEQUEST algorithm. Peptide precursor mass tolerance was set at 10 ppm, and MS/MS tolerance was set at 0.6 Da. Search criteria included carbamidomethylation of cysteine (+ 57.0214 Da) as a fixed modification and oxidation of methionine (+ 15.9949 Da) and SILAC labels [+ 6.02 Da (R) or + 10.008 Da (R) and + 4.025 Da (K) or + 8.014 Da (K)] as variable modifications. Searches were performed with full tryptic digestion and a maximum of 1 missed cleavage was allowed. The reverse database search option was enabled and all peptide data were filtered to satisfy a 1% false discovery rate (FDR). Contaminants, reverse database hits and hits corresponding to one single peptide were removed. Protein ratios were calculated and converted into their log2. Putative interaction partners were selected when their ratios were above the cut-off (mean + 1.96 SD) and had been identified in at least two of the three replicates unless otherwise indicated. The mass spectrometry proteomics data have been deposited to the ProteomeXchange Consortium via the PRIDE partner repository with the dataset identifier PXD023086.

### SUnSET, Global Protein Synthesis Measurement

Cos-7 cells were transfected with empty Flag and UBCv1, and further mock-infected or BPP30GFP-infected at several times. Additionally, cells were treated or non-treated with the protein synthesis inhibitor cycloheximide (CHX). Then, cells were pulsed with 10 μg/ml of puromycin at 37°C and 5% CO_2_ for 10 min. After two washes with fresh complete medium, cells were incubated for 50 min at 37°C and 5% CO_2_ (chase). After pulse/chase experiments, cells were processed for WB or IF.

### m7GTP–Sepharose Pull-Down Assay

Vero cells were infected with Ba71V and harvested at several times post-infection. Cells were washed once with ice-cold PBS and lysed with lysis buffer (100 mM NaCl, 20 mM Tris-HCl pH 7.4, 0.5% NP-40, 1 mM NaF, 5 mM MgCl_2_, and 5 mM NaVO3 with 1 X protease inhibitors [Roche] in water) for 1 h at 4°C. After centrifugation (13,000 × *g* for 30 min), supernatants were incubated with Sepharose-4B beads (Jena Bioscience) for 10 min as a pre-clearing step followed by the cap-binding reaction with m7GFP-Sepharose beads (Jena Bioscience) for 2 h at 4°C. Then, m7GTP-Sepharose beads were washed 6 times with lysis buffer, incubated with 1 mM GTP, and then resuspended in sample buffer and subjected to SDS-PAGE and WB analysis.

### Statistical Analysis

The experimental data were analyzed by one-way ANOVA by Graph Pad Prism 5 software. For multiple comparisons, Bonferroni’s correction was applied. Values were expressed in graph bars as mean ± SD of at least three independent experiments unless otherwise noted. A *p* < 0.05 was considered as statistically significant.

## Results

### UBCv1 Expression and Subcellular Localization in Infected Cells

**FIGURE 1:**
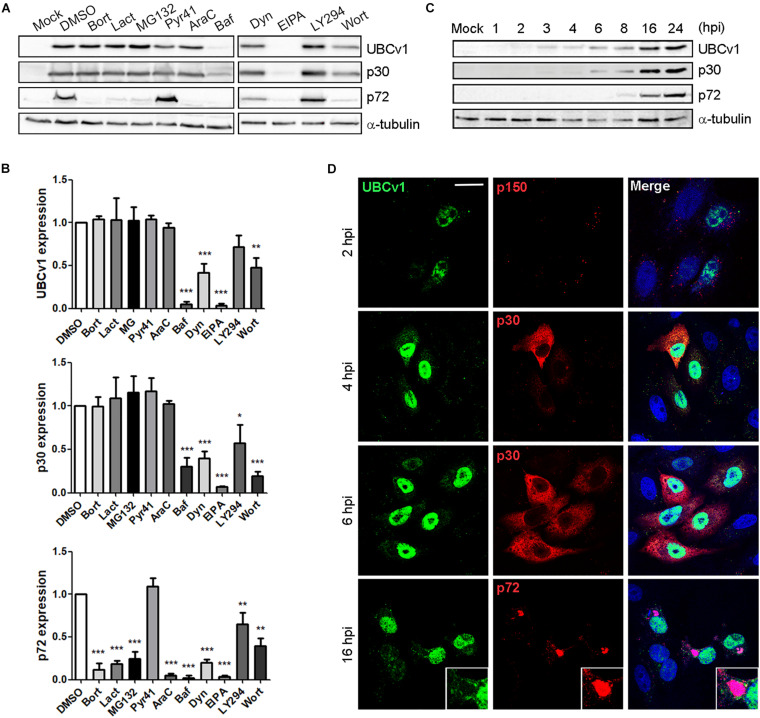
UBCv1 expression at ASFV infection. **(A)** Analysis of UBCv1 expression in infected Vero cells at several times post-infection. Representative western blot (WB) images of viral UBCv1, early p30 and late p72 expression in cells pretreated with Bort, Lact, MG132 (0.5, 20, and 1 μM), AraC (100 μg/ml), Baf, Dyn, EIPA (0.2, 80, and 50 μM), Pyr, Wort, or LY (10 μM) or DMSO and infected with ASFV for 16 hpi. **(B)** Quantification of the bands was corrected to tubulin data, normalized to control, and compared to DMSO. Significant differences are marked with asterisks as indicated (*p < 0.05; **p < 0.01; ***p < 0.001). **(C)** Expression of UBCv1 at several times after infection detected with an α-UBCv1 rabbit antiserum by WB. **(D)** Representative confocal micrographs of the distribution of UBCv1 in infected cells at several times. Cells were labeled for UBCv1 (green) using a polyclonal serum and monoclonal antibodies (red) against the following viral proteins p150 (virions), early p30 (cytoplasm) or late p72 protein (viral factories). Bar = 20μm.

We first characterized UBCv1 synthesis using chemical inhibitors of several infection stages. The impact of these drugs on UBCv1 and other viral protein expression was analyzed by WB. Proteasome inhibitors bortezomib (Bort), lactacystin (Lact), and MG132 did not modify early viral protein p30 nor UBCv1 protein levels. However, these drugs induced a 3–4-fold decrease in late p72 viral protein expression. Pyr-41 did not significantly alter neither UBCv1 expression nor other infection parameters such as p30 and p72 expression (Figures 1A,B). In DNA synthesis inhibitor AraC-treated samples UBCv1 and p30 proteins could be detected, as both are early expressed and independent of viral DNA replication. In contrast, as ASFV late gene expression is dependent on virus DNA replication, we observed a 10-fold reduction of p72 expression levels. We previously reported that ASFV enters cells by a dynamin-dependent and clathrin-mediated endocytosis mechanism (Hernaez and Alonso, 2010). As expected, bafilomycin (Baf) treatment was followed by a 10- and 4-fold reductions in UBCv1 and p30 expression, respectively (Figure 1A). Consequently, late p72 expression also presented a 12-fold decrease. Dynasore (Dyn) inhibited drastically p30 and UBCv1 expression and therefore p72 expression. Treatment with EIPA, Na^+^H^+^ exchanger inhibitor of macropinocytosis (Koivusalo et al., 2010), also entailed an almost complete inhibition of the infection. Previous reports identified that EIPA affects negatively infection not only at early but also at late times of infection (Galindo et al., 2015). PI3K inhibitors LY294002 (LY294) and wortmanin (Wort), decreased early protein p30 and UBCv1 expression as this kinase is involved in post-entry signaling related to early endosomes and subseqeuntly decreased late protein p72 from 1.5- to 2-fold (Figures 1A,B). In conclusion, inhibitor screening also characterized UBCv1 as a very early protein.

In parallel, we also analyzed the expression and subcellular distribution of UBCv1 with a rabbit antiserum generated in our laboratory. Vero cells were infected with ASFV and harvested at various times post-infection. WB analysis showed UBCv1 expression starting at 2 hpi and up to 24 hpi. Indeed, a higher amount of UBCv1 was detected late post-infection times, indicating that this protein accumulates throughout the course of infection (Figure 1C). UBCv1 was found preferentially in the nucleus of infected cells, being also present but to a lesser extent in the cytoplasm late after infection (Figure 1D).

### UBCv1 Acts as an E2 Conjugating Enzyme

**FIGURE 2:**
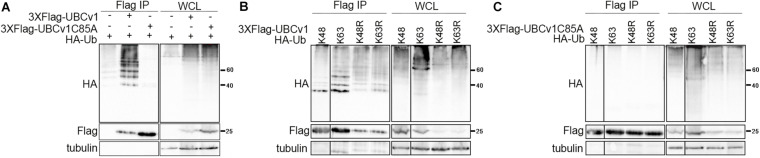
UBCv1 functions as an E2 conjugating enzyme. **(A)** Analysis of the interaction between UBCv1/UBCv1C85A and ubiquitin in an ubiquitylation assay. **(B)** Representative images of the immunoprecipitation of HEK293 co-expressing Flag-UBCv1 plasmid or **(C)** Flag-UBCv1C85A and one of the following HA-Ub variations: HA-UbK48, HA-UbK63, HA-UbK48R, or HAUb-K63R. Spliced and grouped images from blots in boxes in (**A–C)** are shown in full in Supplementary Figure S1. Western blotting against HA and Flag tags was used for detection and whole-cell lysates (WCL) were loaded as transfection rate controls.

Considering that UBCv1 protein shares 48% amino acid identity with the cellular proteins E2 conjugating enzyme G2 and 44.2% with E2R2, we analyzed whether UBCv1 was ubiquitylated in transfected cells and if so, which kind of chains it associated. To address that, we performed an ubiquitylation assay as described in section “Materials and Methods.” Whole-cell lysate (WCL) for wild-type ubiquitin (HA-Ub) resolved as a smear in the upper part of the membrane in all samples (Figure 2 and Supplementary Figure S1). After Flag immunoprecipitation (Flag IP), ubiquitin bands between 40 and 60 KDa were detected with an anti-HA antibody in the presence of UBCv1 but not UBCv1C85A, indicating that UBCv1 was able to associate with wild type ubiquitin (Figure 2A). Next, we performed a similar experiment including ubiquitin mutants K48 and K63, which contain only one lysine residue at positions 48 (HA-UbK48) or 63 (HA-UbK63) as well as mutants R48 and R63 which are unable to form any polyubiquitin chain at Lys48 (HA-UbK48R) or Lys63 (HA-UbK63R). After co-expressing each of these ubiquitin mutants with UBCv1 we detected a smear in WCL samples and a variable ubiquitin pattern after Flag immunoprecipitation (Figure 2B). We found that UBCv1 undergoes K63 and K48 polyubiquitylation. As a negative control, we performed the same experiment using the UBCv1C85A with a point mutation in the catalytic domain. As expected, UBCv1C85A was not ubiquitylated by any mutated Ub after immunoprecipitation experiments (Figure 2C). These data mainly demonstrate the high versatility of UBCv1 to associate with different ubiquitin chains, also indicating the importance of the Cys85 at the catalytic domain for its enzymatic activity.

### UBCv1 Interacts With the Ribosomal Protein RPS23 and They Co-migrate in the Same Ribosomal Fractions

**FIGURE 3:**
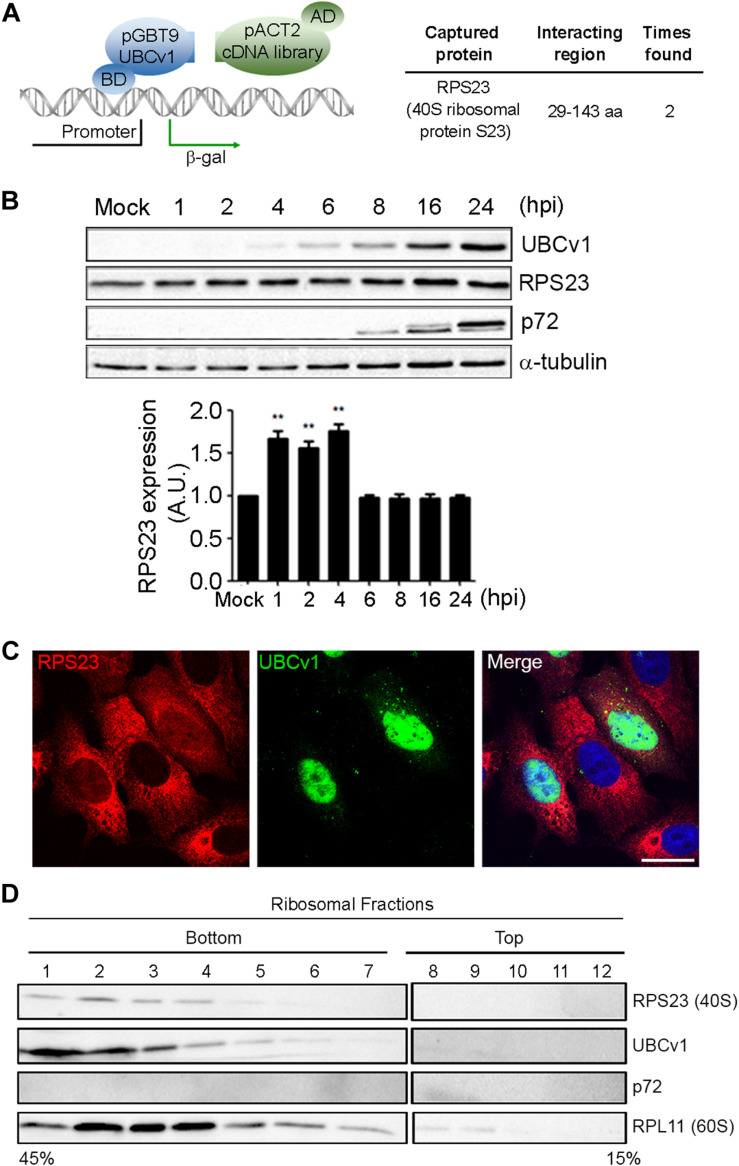
Interaction between viral UBCv1 and cellular RPS23 protein in cellular fractions. **(A)** pGBT9-UBCv1 was used to screen a pACT2 cDNA library from swine alveolar macrophages in a yeast two-hybrid assay. Clones encoding interacting proteins were selected by expression of β-galactosidase (β-gal). The coding sequences contained in the positive clones were amplified, sequenced, and matched in the NCBI database to determine the interacting region with UBCv1. (BD: DNA-binding domain, AD: activating domain). **(B)** RPS23 expression at several times postinfection by WB. Late p72 and UBCV1 proteins were included to confirm the infection and tubulin as load control. Quantification of the bands was corrected with tubulin data, normalized to controls and compared to mock-infected cells. Significant differences are marked with asterisks (**p < 0.01). **(C)** Representative confocal microscopy of UBCv1 (green) and RPS23 (red) detected in infected cells at 16 hpi. Bar = 20 μm. **(D)** Representative WB images of the presence of UBCv1 in cellular ribosomal fractions. RPS23 and UBCv1 proteins were found in the same fractions. We used RPL11 as a fractionation control and late viral protein p72 as control of specificity.

Then, we analyzed potential host interactions through the yeast two-hybrid assay (YTH) to find the 40s ribosomal subunit protein S23 (RPS23) as an UBCv1 interacting partner (Figure 3A). Given that UBCv1 was shown to interact with K48-Ub chains, we studied whether UBCv1 could affect the stability of RPS23. First, we looked at the expression profile of this protein upon ASFV infection by WB at different time points. ASFV-infected cells showed significantly increased RPS23 relative expression levels at early time points (1, 2, 4 hpi) followed by a drop to mock-infected cells levels at later post-infection times (6–24 hpi) (Figure 3B). Regarding RPS23 subcellular localization, we observed a shift from the cytoplasm to the nucleus of ASFV infected cells that was absent in non-infected Vero cells (Figure 3C). We also analyzed the presence of this viral protein in cellular ribosomal fractions. In the process of protein translation, the small (40s) and the large (60s) ribosomal subunits associate with the mRNA. Polysomes are complexes consisting of multiple ribosomes simultaneously translating a single mRNA into a polypeptide chain and the ribosomes move along the mRNA as translation elongation occurs (Jan et al., 2016). We isolated ribosomal subunits from polysomes, monosomes, and messenger ribonucleoproteins (mRNPs). Samples obtained after ribosome fractionation were examined by WB for the presence of UBCv1, RPS23, and the 60S subunit RPL11 protein as a control of the ribosomal fractionation. Our results showed that viral UBCv1 and RPS23 bands co-migrated in the same ribosomal fractions (Figure 3D, lanes 1–6). As expected, an unrelated viral protein, p72, did not present the same pattern and lacked ribosome association, confirming the specificity of this observation.

### ASFV UBCv1 Binds Cellular Translation Initiator Factor eIF4E

**FIGURE 4:**
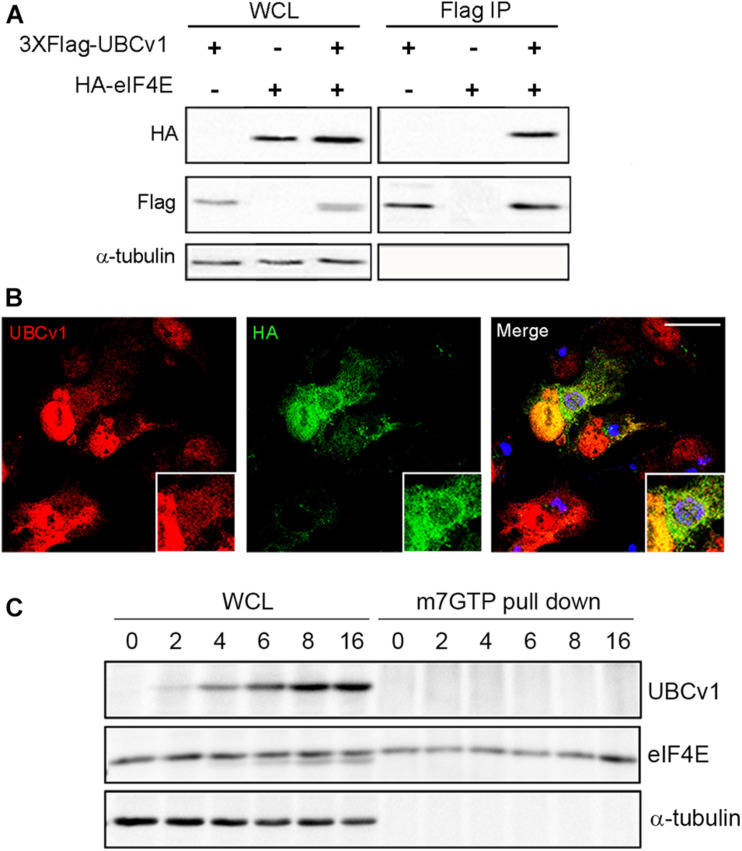
ASFV UBCv1 binds cellular translation factor eIF4E. **(A)** IP assay of UBCv1 protein and eIF4E, analyzed by western blot. The interaction between both proteins was detected using α-FLAG and α-HA antibodies and transfection rates and expression checked in WCL samples. **(B)** Representative confocal microscopy images of cells transfected with HA-eIF4E for 12 h and infected with Ba71V for 16 h. Colocalization for UBCv1 (red) and HA (eIF4E, green) was observed as a yellow signal. VFs of infected cells stained for viral protein p72 (blue). Bar = 20μm **(C)** Cap-complex immunoprecipitation using the cap-analog m7GTP-Sepharose of Ba71V infected cells. eIF4E bound to the cap-complex but not UBCv1, which bound eIF4E independently from the cap-complex.

We also analyzed UBCv1 interactors by mass spectrometry analysis using Stable Isotope Labeling by Amino acids in Cell culture (SILAC) as described in section “Materials and Methods.” Among the hits, we identified the eukaryotic translation initiation factor eIF4E which serves as a scaffolding protein between the mRNA and the small ribosomal subunit 40S, and the E3 ligase Cullin4B (Cul4B). We first validated the interaction with eIF4E through immunoprecipitation experiments. Cells were co-transfected with pcDNA-3XFlag-UBCv1 and pcDNA3-HA-eIF4E or single transfected with each individual plasmid as negative controls. eIF4E immunoprecipitated UBCv1 (Figure 4A), while no band was detected in our control samples in these experiments. The interaction was further confirmed by confocal microscopy using specific antibodies against HA (eIF4E) and UBCv1. Colocalization between these proteins was observed with an intense overlapping yellow signal in the nucleus (Figure 4B).

Next, we analyzed a possible binding of UBCv1 to the cap-dependent translation complex eIF4F through an eIF4E interaction. The potential presence of UBCv1 in the cap complex was investigated using the cap analog m7GTP (7-methylguanosine 5′-triphosphate) immobilized to Sepharose in a pull-down assay. m7GTP–Sepharose was incubated with protein lysates from ASFV Vero-infected cells at the indicated time points. Bound eIF4E was isolated with m7GTP and resolved by SDS-PAGE. In the WB, eIF4E was bound to the cap-complex but UBCv1 was not detected, although it was present in the whole-cell lysates as a control (Figure 4C). These results indicate that the viral protein UBCv1 does not form part of the cap-complex when binding eIF4E.

### ASFV Infection Regulates mTOR Signaling and Protein Expression

**FIGURE 5:**
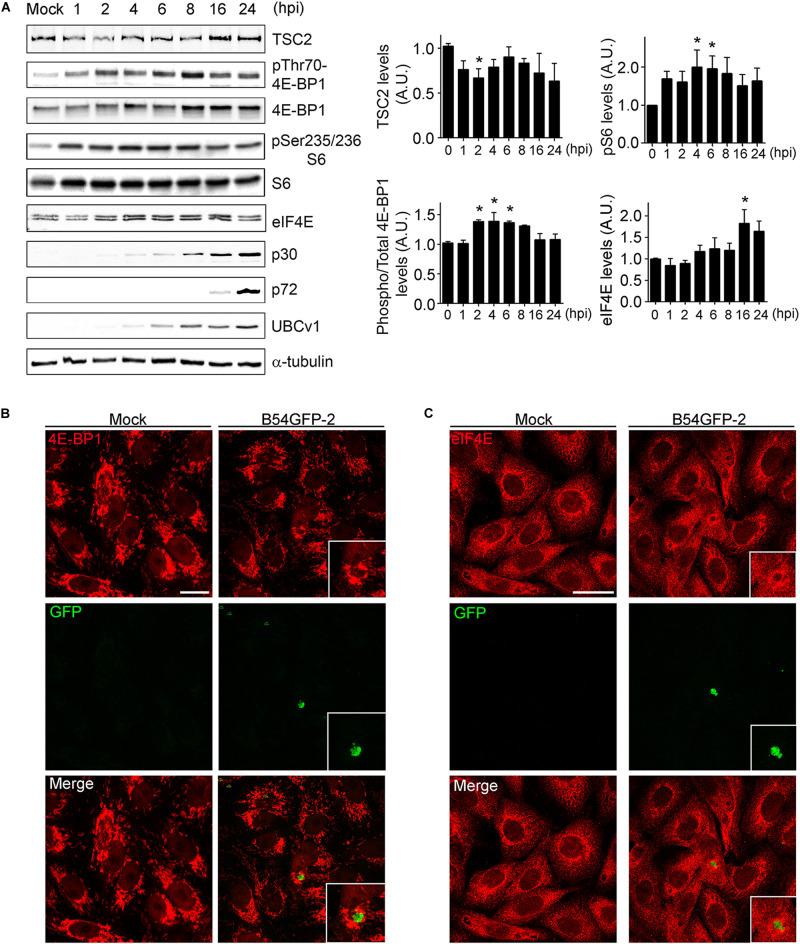
ASFV infection regulates mTOR signaling. **(A)** TSC2, eIF4E, total and phosphorylated 4E-BP1 and S6 expression were analyzed at several times postinfection by WB using specific antibodies. Early p30, late p72, and UBCv1 proteins were included to confirm infection and tubulin as load control. Quantification of the bands was corrected to tubulin data, normalized to control values, and compared to uninfected cells (Mock). Significant differences are marked with asterisks (*p < 0.05). **(B)** Immunofluorescence (IF) to analyze 4E-BP1 expression (red) in B54GFP-2 (green) infected cells at 16 hpi localized in VFs. Bar = 20μm. **(C)** Immunofluorescence to analyze eIF4E expression (red) in B54GFP-2 (green) infected cells at 16 hpi localized in VFs. Bar = 20μm.

After studying the viral UBCv1 interaction with the cap-dependent host translation factor eIF4E, we investigated if ASFV infection could modulate mTORC1 signaling and protein expression. It is well known that mTOR activity can regulate the assembly of the eIF4F complex inducing 4E-BPs inactivation by hyperphosphorylation. Inactivation of 4E-BP prevents this inhibitory factor to bind and hijack the eIF4E factor to inhibit translation (Bhandari et al., 2001; Richter and Sonenberg, 2005). First, we analyzed the expression of TSC2 (tuberous sclerosis complex 2), a repressor of mTORC1 pathway at ASFV infection. We detected a significant decrease of TSC2 levels by WB at 2 hpi compared to mock-infected cells followed by a recovery of basal levels after 6 h (Figure 5A). Then, we analyzed the phosphorylation status of 4E-BP1 and S6, as substrates of mTOR activity, and we observed a significant increase of 4E-BP1 and S6 phosphorylation after 2 hpi, which was not evident after 6 h of infection (Figure 5A), both facts supporting an early activation of mTOR.

We also analyzed the subcellular distribution of 4E-BP1 in mock and infected cells by confocal microscopy. Cells were infected with B54GFP-2 (Hernaez et al., 2006) to detect viral factories (VFs) and then stained for 4E-BP1 (red). In uninfected cells, 4E-BP1 expression was mainly cytoplasmic around the cell nucleus. However, 4E-BP1 expression is redistributed around ASFV VFs in infected cells (Figure 5B). We studied protein expression and subcellular distribution of eIF4E in mock and infected cells. We detected an increase of eIF4E at 16 hpi compared to mock-infected cells by WB (Figure 5A).

Localization of eIF4E expression was also detected by IF assay. Cells were infected with B54GFP-2 (Hernaez et al., 2006) to detect viral factories (VFs), and then stained for eIF4E. In mock cells, eIF4E expression was mainly cytoplasmic with a certain punctate pattern. However, a high expression of eIF4E was observed surrounding ASF VFs, as typically occurs with several host translation factors in infected cells (Figure 5C). These results indicate that ASFV may alter the mTOR signaling pathway during infection actively recruiting the host translation machinery to the VFs.

### UBCv1 Interacts With the Cullin-RING E3 Ubiquitin Ligase Cul4B

**FIGURE 6:**
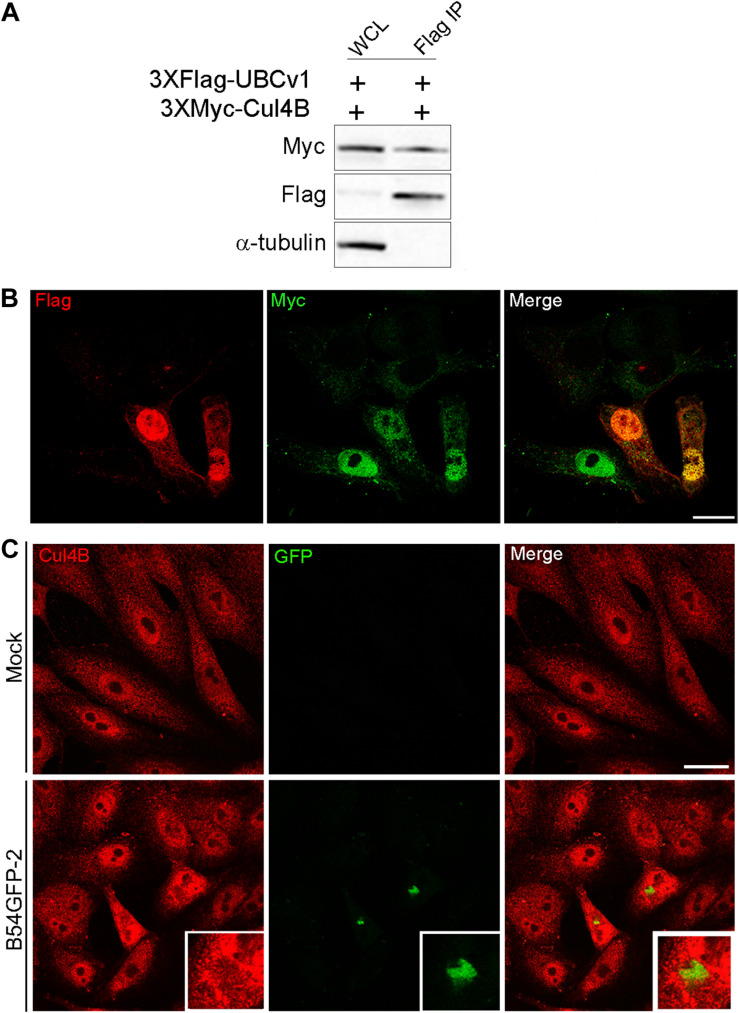
UBCv1 viral protein interacts with Cul4B ligase. **(A)** IP assays of the co-transfection of UBCv1 and Cul4B. The interaction between both proteins was detected using α-Flag and α-Myc. **(B)** Representative confocal microscopy of Vero cells transfected with myc-Cul4B and 3XFlag-UBCv1 for 12 h. Colocalization was observed at the nucleus using α-Flag (UBCv1, red) and α-Myc (Cul4B, green) antibodies. **(C)** Cul4B expression in mock or B54GFP-2 infected cells at 16hpi. Cells were labeled for Cul4B (red) and viral p54GFP (green) that is localized in VFs with some colocalization spots for Cul4B in VFs. Bar = 20μm.

Another interacting partner of UBCv1 found in the SILAC analysis was the cellular E3 ligase Cullin 4B (Cul4B). Cul4B has been recently identified to play an important role in regulating TSC2 and mTOR signaling in some brain diseases (Wang et al., 2013), and it is also able to degrade 4E-BP2 eukaryotic translation initiation factor (Kouloulia et al., 2019). Then, E3 ligase Cul4B could be a potential interacting partner of ASFV UBCv1. To confirm this interaction, Vero cells were co-transfected with constructs expressing FLAG-UBCv1 and Myc-Cul4B. Interestingly, we were able to detect both proteins in the immunoprecipitated material, thus corroborating the UBCv1 binding to Cul4B (Figure 6A).

Additionally, subcellular localization of these proteins was further confirmed through confocal microscopy, and both proteins were mainly observed colocalizing in the nucleus of co-transfected cells expressing FLAG-UBCv1 and Myc-Cul4B (Figure 6B). Then, we studied the subcellular distribution of Cul4B in infected and mock-infected cells. While protein expression was mainly observed in the nucleus and the cytoplasm of control cells, Cul4B protein levels were increased in infected cells and accumulated in the nucleus and around the VFs, similar to what was shown for other host translation-related factors (Figure 6C).

### Regulation of Protein Synthesis Under UBCv1 Expression

Then, we analyzed UBCv1 ability to alter global host protein expression using the SunSet method as previously described (Schmidt et al., 2009). It allowed us to monitor and quantify global protein synthesis in individual mammalian cells by immunofluorescence. Cos-7 cells were transfected with the pcDNA4/TO-3XFlag-UBCv1 plasmid or infected with ASFV BPP30GFP at several times. As an internal control, we used the protein synthesis inhibitor cycloheximide. After 24 h, cells were treated with 10 mg/ml of puromycin. Puromycin is incorporated into the elongating peptides and released from the ribosome as puromycin-labeled peptides. A 10-min pulse, followed by a 50-min chase allowed puromycin-labeled proteins to be detected by WB and IF using an anti-puromycin (12D10) monoclonal antibody.

**FIGURE 7:**
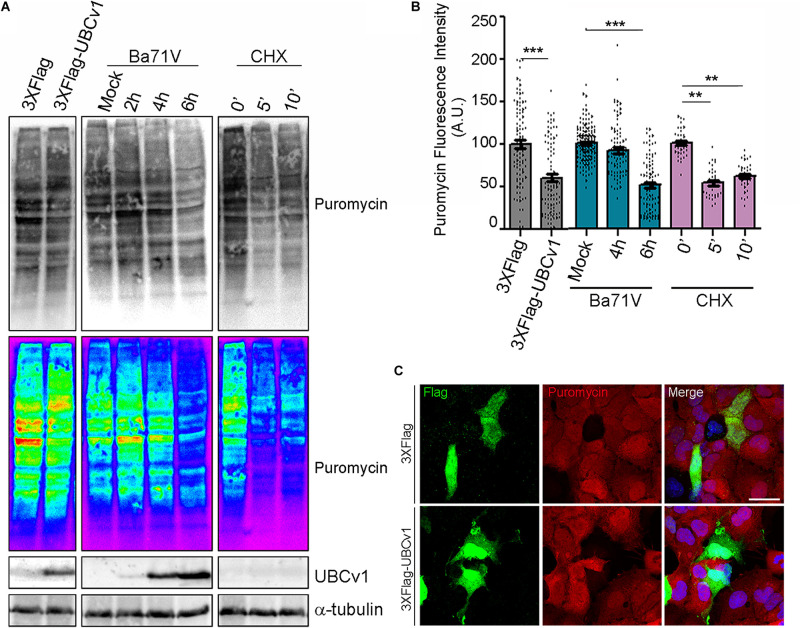
Global inhibition of host protein synthesis. SunSet protein expression assay was analyzed by WB **(A)** and IF **(B,C)**. Cos-7 cells were infected with ASFV-BPP30GFP at the indicated times, transfected with UBCv1 plasmid or treated with cycloheximide (CHX), and then pulsed with puromycin to detect newly synthesized proteins. Puromycin incorporation to the new peptides and UBCv1 expression, were analyzed by western blot **(A)** or IF **(B,C)** using specific antibodies. **(B)** Quantification of puromycin staining by IF in transfected/infected cells or cells treated with cycloheximide. UBCv1 overexpression and ASFV infection after 6 h reduce puromycin incorporation. Graphics depict mean ± SEM of fluorescence values measured with ImageJ and each point represents one single cell fluorescence intensity. Significant differences are marked with asterisks (**p < 0.001, ***p < 0.0001). **(C)** Representative confocal images of Cos-7 cells transfected with pcDNA4/TO-3XFlag or Flag-UBCv1 plasmids. Puromycin reduction in Flag-UBCv1 positive cells indicates inhibition of the host protein synthesis. Bar = 20μm.

Following this strategy, we found that ASFV infection promoted a reduction of puromycin labeling after 6 h by WB analysis thus supporting a reduction of translation in infected cells. Moreover, we observed that UBCv1 overexpression promoted a reduction of puromycin-labeled proteins over time (Figure 7A). This result was further confirmed through confocal microscopy. Puromycin expression was quantified in transfected or infected cells and detected by using either α-Flag antibody or GFP expression, respectively (Figure 7B). A reduction in puromycin expression levels was quantified in several transfected cells as shown in Figure 7B. This decrease of puromycin staining in the presence of UBCv1 was equivalent to that observed after 6 h of ASFV infection (Figure 7B). Figure 7C shows that Flag-UBCv1 overexpression reduced puromycin staining in transfected cells (lower panel) compared to cells transfected with a Flag empty plasmid (upper panel, Figure 7C). Collectively, these results indicate that viral protein UBCv1 may be implicated in the shut-off of protein synthesis induced by ASFV in infected cells.

## Discussion

Ubiquitylation is a posttranslational modification that controls almost every cell process (Komander and Rape, 2012). Some viral proteins can interact with cellular E3 ubiquitin ligases and induce their ubiquitylation. This has a positive effect on the replication abilities of DNA viruses such as herpesvirus (Tran et al., 2010), poxviruses (Teale et al., 2009), and adenoviruses (Gupta et al., 2013). Similarly, ASFV also requires the ubiquitin-proteasome system (UPS) to replicate in infected cells (Barrado-Gil et al., 2017). Some large DNA viruses like herpesvirus and poxviruses also encode their E3 ligases (Ishido, 2010; Boname and Lehner, 2011) or their deubiquitinases DUBs to evade the host innate immune system and promote viral replication (Bailey-Elkin et al., 2017; Jahan et al., 2020).

In contrast, our candidate gene to study was ASFV gene *I215L* encoding an ubiquitin-conjugating enzyme, UBCv1. The scientific interest of this protein is high, given the fact that is the only E2 conjugating enzyme encoded by a virus described to date (Hingamp et al., 1992). Viral genome expression is regulated in a temporal fashion with early expressed proteins and others synthesized after viral replication or late proteins. Early viral proteins are responsible for exploiting the host cell machinery at the beginning of the infection. It was previously described that this protein is synthesized before viral replication (Yanez et al., 1995; Freitas et al., 2018) and it continues to be synthesized until late times after infection (Hingamp et al., 1995). Using a panel of inhibitors, we could determine the infection stage relevant for UBCv1 expression. So, we determined that UBCv1 expression occurred immediately following dynamin and clathrin-mediated cell entry but before acid-dependent decapsidation or EIPA inhibition. Also, inhibition of early endosome signaling, reduced expression. This indicated that UBCv1 is a very early viral protein synthesized upon cell entry with a similar expression profile to early p30 protein. UBCv1 expression was independent of viral DNA replication and progressively accumulated at late post-infection times. Previous reports have described UBCv1 localization limited to the cytoplasm of infected cells (Hingamp et al., 1995), in contrast to others describing its localization both in the nucleus and VFs (Freitas et al., 2018). We found UBCv1 distributed predominantly in the nucleus of infected cells at early time points, while at late stages of infection, it could be detected in the cytoplasm as well. This suggests that UBCv1 could dynamically shuttle between the nucleus and the cytoplasm and change along with infection. Also, and similar to p30 (Afonso et al., 1992), UBCv1 could not be detected in the VFs of ASFV infected cells.

Previous studies revealed UBCv1 conjugating activity (Freitas et al., 2018), though UBCv1 *in vivo* substrate(s) for this viral enzyme are not known yet. As previously shown, recombinant UBCv1 can be self-ubiquitinated *in vitro* and it can also ubiquitinate histones as well as the ASFV virion core-shell polyprotein pp62 (pp62) (Simon-Mateo et al., 1997). Given its relevance, we designed several strategies for analysis of this protein function.

E2 conjugating enzymes are central players of the enzymatic process of ubiquitylation, though are often presented as simple carriers of ubiquitin. However, E2s can determine when and how a specific target would be modified by Ub (Stewart et al., 2016). Our results showed that UBCv1 was active as an ubiquitin-conjugating enzyme with high versatility by associating with several classes of polyubiquitin chains and it is dependent on its catalytic domain.

We have characterized an interaction between UBCv1 and the 40s ribosomal protein S23 (RPS23) that was corroborated by ribosome fractionation. We did not detect RPS23 degradation as a result of this binding, but also it is conceivable that UBCv1 could act preventing potential mRNAs or other factors from binding to this position thus resulting in subsequent ribosomal stalling or pausing. In general, the consequences of ubiquitylation depend on chain topology, timing, and reversibility of the reaction, enzyme or substrate localization, and finally on interactions between E3 ligases and their effectors (Kim et al., 2011).

Proteins are not built at ribosomes at a constant rate. In fact, there are many examples of proteins that stall at the ribosome exit tunnel. It is thought this stalling is how cells can control the expression of proteins and this system could be hijacked by viral proteins (Walsh et al., 2013). Hence, the overall cell capacity for protein synthesis would be reduced and protein quality control would degrade stalled proteins. UBCv1 and RPS23 were expressed both in the nucleus and cytoplasm of infected cells, indicating that the interaction could be possible in both localizations. However, UBCv1 co-migrated in the same ribosomal fractions as RPS23, while unrelated viral protein p72 did not show that profile.

Translation initiation is one of the most regulated steps of gene expression. The eukaryotic initiation factors (eIFs) play an essential role in the recruitment of mRNA to the ribosome and therefore, they are potential targets for viruses (Montero et al., 2015). We have identified the interaction of UBCv1 with eIF4E through mass spectrometry and characterized that eIF4E increases its expression upon viral infection. UBCv1 was able to bind to eIF4E and induced this factor overexpression, similarly to Epstein-Barr virus latent membrane protein 1 (LMP1) resulting in increased protein synthesis (Zhao et al., 2014). Cap-dependent translation starts with eIF4E binding to the mRNAs cap, nucleating the translation initiation complex with eIF4G and eIF4A. 4E-BPs inhibit the formation of this complex by competing for binding to eIF4E and preventing eIF4F formation and initiation (Merrick and Pavitt, 2018). Our results indicate that eIF4E interacts with UBCv1 apart from the cap-complex but it does not prevent the assembly of eIF4E for complex formation and translation initiation.

Similar to other viruses, we also found initiation factors eIF4E and 4E-BP1 reorganized around VFs in ASFV infected cells (Katsafanas and Moss, 2007; Walsh et al., 2008). This would aid recruiting of ribosomes and the cap-dependent translation machinery to the viral mRNAs for the synthesis of late viral protein and although the implication of eIF4E in ASFV replication and protein synthesis has been previously described (Castello et al., 2009; Sanchez et al., 2013), this is the first direct interaction reported between this factor and a given ASFV protein.

SARS-CoV-2 Orf10 interacts with multiple members of a Cullin2 (CUL2) RING E3 ligase complex that targets substrates for degradation (Gordon et al., 2020). Interestingly, we also found that UBCv1 binds to Cullin RING ligase Cul4B. Cul4B plays an important role in regulating TSC2 and mTOR signaling (Wang et al., 2013) and its abundance control 4E-BP2 eukaryotic translation initiation factor (Kouloulia et al., 2019). Upon phosphorylation by the mammalian target of Rapamycin (mTORC1) kinase, 4E-BPs reduce binding affinity to eIF4E (Pause et al., 1994) and constitute another regulation mechanism. Based on this regulation, we observed that TSC2, a repressor of the mTORC1 pathway, was transiently inhibited upon ASFV infection at early time points (2 hpi) followed by a recovery to basal levels after 6 hpi. An early and transient increased phosphorylation 4E-BP1 and S6 supports early mTORC activation followed by translation shut-off thereafter. To summarize, we found an inhibition of protein synthesis under transient UBCv1 expression levels similar to ASFV infection, pointing out the relevance of this protein in the control of host translation.

In conclusion, our studies determined that UBCv1 is a multifunctional protein that can bind more than one component of the host translation machinery, highlighting the relevance of ASFV in the regulation of host protein translation.

## Data Availability Statement

The mass spectrometry proteomics data have been deposited to the ProteomeXchange Consortium via the PRIDE partner repository with the dataset identifier PXD023086.

## Author Contributions

CA, EN-V, and CM: conceptualization and supervision. LB-G, AP, IG, RM-M, JU, and EN-V: methodology. IG, MÁC-G, and RM-M: validation. MÁC-G, IG, and JU: formal analysis. LB-G, AP, RM-M, and JU: investigation. CM and EN-V: resources. IG, CM, and EN-V: data curation. CA, LB-G, and AP: writing – original draft preparation. CA: project administration and funding acquisition. All authors: writing – review and editing.

## Conflict of Interest

The authors declare that the research was conducted in the absence of any commercial or financial relationships that could be construed as a potential conflict of interest.
