# Supplementary figures and images for: African Swine Fever Virus Ubiquitin-Conjugating Enzyme Interacts With Host Translation Machinery to Regulate the Host Protein Synthesis
Source: Front Microbiol. 2020 Dec 15;11:622907. doi: 10.3389/fmicb.2020.622907 (PMC7771050; doi:10.3389/fmicb.2020.622907)

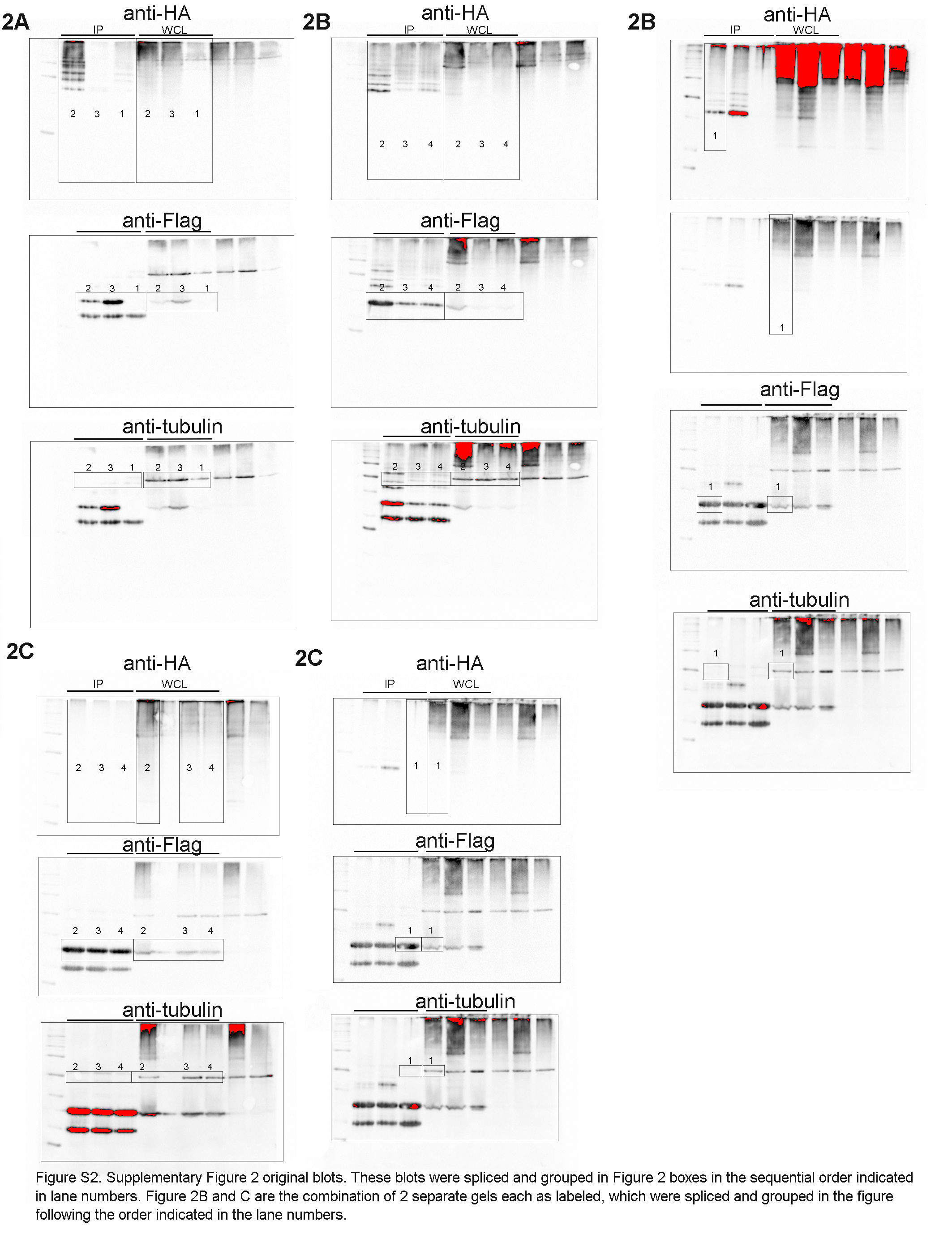

Supplement: Supplementary file 1 [file Image_1.TIF]
